# Overexpressing HPGDS in adipose-derived mesenchymal stem cells reduces inflammatory state and improves wound healing in type 2 diabetic mice

**DOI:** 10.1186/s13287-022-03082-w

**Published:** 2022-08-03

**Authors:** Long Ouyang, Daojing Qiu, Xin Fu, Aiping Wu, Pengyuan Yang, Zhigang Yang, Qian Wang, Li Yan, Ran Xiao

**Affiliations:** 1 Research Center of Plastic Surgery Hospital, Chinese Academy of Medical Sciences & Peking Union Medical College, Beijing, People’s Republic of China; 2 Suzhou Institute of Systems Medicine, Suzhou, Jiangsu People’s Republic of China; 3 Key Laboratory of Infection and Immunity of CAS, CAS Center for Excellence in Biomacromolecules, Institute of Biophysics, Chinese Academy of Sciences, Beijing, People’s Republic of China

**Keywords:** Hpgds, Diabetic wound, ADSC, Inflammation regulator, Healing

## Abstract

**Background:**

In diabetes, delayed wound healing was considered as the result of excessive recruitment and retention of pro-inflammatory cells and factors. Hematopoietic prostaglandin D synthase (HPGDS) was identified from differently expressed genes of diabetic human foot skin. HPGDS is responsible for the production of prostaglandin D2 (PGD2), an inflammatory mediator. Therefore, we aim to explore whether HPGDS could be a therapeutic target in the diabetic wound (DW).

**Method:**

In this study, we compared gene expression profilings of diabetic human foot skin and non-diabetic human foot skin from the Gene Expression Omnibus database. We detected the characteristics of immune components in diabetic mice wound and investigated the role and underlying mechanism of the differently expressed *Hpgds* for the diabetic wound healing. For in vivo studies, we engineered ADSC to overexpress *Hpgds* (ADSC^Hpgds^) and evaluated its effects on diabetic wound healing using a full-thickness skin wound model. For in vitro studies, we evaluated the role of ADSC^Hpgds^ conditioned medium and PGD2 on Lipopolysaccharide (LPS) induced macrophage.

**Results:**

*Hpgds* was significantly down-regulated in type 2 diabetic mice wound and its deficiency delayed normal wound healing. ADSC^Hpgds^ accelerated DW healing by reducing neutrophil and CD8T cell recruitment, promoting M2 macrophage polarization and increasing the production of growth factors. ADSC^Hpgds^ conditioned medium showed superior capability in promoting M2 macrophage transition than conditioned medium derived from ADSC alone.

**Conclusion:**

Our results demonstrated that *Hpgds* is required for wound healing, and ADSC^Hpgds^ could accelerate DW healing by improving anti-inflammatory state and normalizing the proliferation phase of wound healing in mice. These findings provide a new insight in the therapeutic strategy of diabetic wound.

**Supplementary Information:**

The online version contains supplementary material available at 10.1186/s13287-022-03082-w.

## Introduction

Wound healing can be delayed and is prone to infection in the patient with type 2 diabetes. Several issues, such as hard-to-control infections, neurological and vascular disorders, and interrupted transition from the inflammatory to the proliferative phase, disturb the process of repair in type 2 diabetic wound (DW) [1–4]. Currently, the main clinical treatment strategies for DW are surgical debridement, wound care, anti-inflammatory treatment, and methods to improve blood supply and provide an environment suitable for tissue regeneration [5]. Moreover, several animal experiments using the growth factors combined with traditional approaches [6–10], including platelet-derived growth factor (PDGF), vascular endothelial growth factor (VEGF), human epidermal growth factor (hEGF), basic fibroblast growth factor (bFGF), and insulin-like growth factor (IGF), showed beneficial effects. However, the therapeutic effects of most clinical treatments are limited.

Mesenchymal stem cells (MSCs) therapy has been reported to facilitate the healing of type 2 DW, in which MSCs can accelerate neovascularization and replenish the extracellular matrix (ECM) through producing growth factors [11, 12]. However, in type 2 DW, the prolonged inflammation is thought to increase the levels of metalloproteinases and other proteases that destroy composition of extracellular matrix (ECM) and growth factors. Since delayed DW healing is thought to be due to impaired macrophages, neutrophil functions, and persistent inflammation [13, 14], both capacities of modulating local inflammation and enhancing tissue regeneration should be restored in the healing environment to achieve an efficient repair of DW. Therefore, the critical inflammation regulatory factor in DW needs to be identified and used to regulate inflammatory state and strengthen the function of MSCs.

In the current study, we compared gene expression profilings (GSE 68,183) of diabetic human foot skin (DFS) and non-diabetic human foot skin (NFS) from the Gene Expression Omnibus (GEO) database [15], and identified differently expressed hematopoietic prostaglandin D synthase (HPGDS). We furtherly found that topical downregulation of *Hpgds* by *Hpgds* short-interfering RNA (siRNA) could delay normal wound healing. HPGDS is a cytosolic protein, mainly located in antigen-presenting cells and mast cells [16], that converts prostaglandin H2 (PGH_2_) into prostaglandin D2 (PGD_2_). 15-Deoxy-Δ^12,14^-prostaglandin J_2_ (15d-PGJ_2_) is the terminal metabolite of PGD_2_ and can be secreted by many cells including mast cells, T cells, platelets, and macrophages. PGD_2_/15d-PGJ_2_ reportedly exerts anti-inflammatory effects by impeding the infiltration of dendritic cells and neutrophils [17] or inducing M2 macrophage polarization [18]. Previous studies showed that PGD_2_ represented an anti-inflammatory response during acute lung injury progression [19], and *Hpgds* deficiency enhanced the inflammatory response in the oviduct [20]. Therefore, we propose that decreased *Hpgds* level is an important reason for delayed DW healing, to upregulate topical *Hpgds* expression could adjust the inflammation state and accelerate DW healing.

Thus, we investigated the inflammation state of DW and assessed the intervention effects of *Hpgds* overexpressed in human adipose-derived mesenchymal stem cells (ADSCs) seeded in VitroGel. ADSCs were chosen as the seed cell because they can be harvested easily and can differentiate into various cell types. In addition, ADSCs possess general anti-inflammation and neoangiogenic effects, and secrete diversified growth factors [21]. ADSCs can also be engineered as sustained sources for the local release of certain gene products. However, the local environment of DW is extremely inhospitable for transplanted cells. It has been reported that arginylglycylaspartic acid (RGD)-coupled hydrogel scaffold (VitroGel 3D-RGD) could provide an appropriate physiochemical microenvironment to promote MSC adhesion and viability [22]. Besides, the physical separation could protect transplanted cells from the host immune system in the early stage. In our study, roles of *Hpgds* at DW sites were investigated by overexpressing *Hpgds* in ADSCs encapsulated in VitroGel 3D-RGD.

## Materials and methods

### Methodology for the bioinformatic analysis of the microarray data

The gene expression profile GSE 68,183 was identified following a search of the Gene Expression Omnibus (GEO) (GEO query 2.40.0), which contained microarrays of diabetic human foot skin (DFS) and non-diabetic human foot skin (NFS). The differentially expressed mRNAs were screened by the “limma” package of R language. The overlapped differentially expressed genes (DEGs) in the network were analyzed to explore the biological processes involved by enrichment and pathway analysis by the “clusterProfiler” package of the R language. The key molecular was identified in the molecular protein–protein interaction (PPI) network among DEGs.

### Immune cell components evaluation

Seq-ImmuCC is a computational model used to predict immune cell constitution from the RNA-Seq data [23], which includes three key steps. First, wound tissues were obtained from diabetic and normal mice at 0 h, 8 h, 2 days, 6 days, 10 days, 14 days, and total RNA were extracted for RNA-Seq. Second, 10 main immune cells, B cells, CD4 T cells, CD8 T cells, macrophages, monocytes, neutrophils, mast cells, eosinophils, dendritic cells, and natural killer cells were clustered. Third, based on the input signal matrix constructed by 511 signature genes, the support vector regression (SVR) method was applied to deconvolve the target gene expression information for evaluating the proportions of immune cells.

### Isolation and culture of human ADSCs

Adipose tissue was collected from two patients undergoing liposuction in Plastic Surgery Hospital. Both donors were around 40 years old, similar height and weight, and had no other systemic diseases. The written informed consents were given from the donors and the study was approved by the Ethics Committee of the Plastic Surgery Hospital. Adipose tissue was incubated with type I collagenase solution, and its surface markers were analyzed using MSC analysis Kits (BD Biosciences, Franklin Lakes, NJ, at passage USA). Then the cells were maintained in Mesenchymal Stem Cell (MSC) Medium (ScienCell, Carlsbad, CA, USA) and used in passages 3–5 (P3–5). Therefore, ADSCs from two donors were independently used in the following experiments.

### Hpgds vectors and hADSC transduction

The open reading frame (ORF) of *Hpgds* was inserted into PCDH-CMV-MCS-EF1-copGFP (System Biosciences, Mountain View, CA), which can encode full-length *Hpgds* (PCDH-Hpgds-GFP). hADSCs (5 × 10^6^ cells) were infected by lentivirus (3 × 10^8^ Tu/ml) containing PCDH-Hpgds-GFP or PCDH-GFP vectors with 20 ug/ml of polybrene transfection reagent (Thermo Fisher Scientific, Waltham, MA, USA) for 6 h. After that the cells were cultured in fresh culture medium for 72 h. These hADSCs overexpress *Hpgds* (hADSC^Hpgds^) or only GFP in the control group (hADSC^GFP^). To avoid interference with immunofluorescence staining, expression lentiviral vector PCDH-Hpgds without GFP was used in vivo. Hpgds open reading frame (ORF) sequence was as follows:

$$\begin{aligned} & {\text{ATGAGCCAAGGCAAACCCTTGCTTCCCACCCTGCCCCAGCCAAAAAGAAAGAAA}} \\ & {\text{GAAAAGAGAGAAAATGTCCCCACAGCATTTCCTAGAAGCCAATCTTATGAAGGA}} \\ & {\text{ATTTTCTTAACTATGGTTCCCTCTTCTTACGTGACACTATCTTGTGTCAAGTTGACA}} \\ & {\text{AAACTCAAACGAAAACCCAACCAAGATATCAACCAACCAAGCTATGGAACCAGG}} \\ & {\text{TGA}}{.} \\ \end{aligned}$$

### Cell proliferation and cell migration assays

The proliferation and migration capability in vitro of hADSC and hADSC^Hpgds^ were detected. Cell counting Kit-8 was applied to analyze proliferation (CCK-8, Beyotime, Shanghai, China). Absorbance at 450 nm was measured by EnSpire® Multimode Plate Reader (PerkinElmer, Waltham, MA, USA). According to the absorbance values, proliferation curves of the detected cells were plotted using GraphPad Prism 7 (GraphPad Inc., San Diego, CA, USA). About 5 × 10^5^ cells were added to each well of the plate. When confluence reached 80%, three longitudinal lines were drawn evenly on the surface with a sterile micropipette tip. The time course of cell migration was recorded by light microscope (Leica DM3000) and measured with ImageJ software.

### Adipogenic and osteogenic differentiation assay

For adipogenic differentiation, cells were cultured in mesenchymal stem cell culture medium (ScienCell, San Diego, CA, USA) in 6-well plates until confluence reached 100%, then adipogenic medium (AM, medium, Cyagen Biosciences, Santa Clara, CA, USA) was used to induce adipogenic differentiation for 14 days and followed by oil red O staining. For osteogenic differentiation, when the cell confluence reached 60–70%, osteogenic medium (OM, basal medium, Cyagen Biosciences) was applied for osteogenesis differentiation for 21 days and followed by alizarin red staining (Cyagen Biosciences).

### Flow cytometry

The cell density of hADSC^Hpgds^ and hADSC^GFP^ was adjusted to 5 × 10^6^ cell/ml. Samples were incubated with CD44, CD73, CD90, CD105 antibodies (Human MSC Analysis Kit, BD Biosciences) for 30 min. Then 1 ml PBS was added to each tube to wash away excess antibody and centrifuged for 5 min. A FACS Aria II flow cytometer (BD Biosciences) was used to detect the cell surface markers. The results were analyzed using FlowJo 10 software (Ashland, OR, USA).

### Animal and the DW model procedure

The animal experiments were approved by the Ethics Committee of the Plastic Surgery Hospital. 105 male BALB/c mice were obtained from Vital River (Vital River Laboratory, Beijing, China). Their ages range from 6 to 8 weeks, with weight ranging from 18 to 22 g. All mice were given a high-fat diet, which contained 34.9% W/W fat (60% of total calories), 26.2% W/W protein (20% of total calories), and 26.3% W/W carbohydrate (20% of total calories) [24]. After one week adaption, five consecutive days of streptozotocin (50 mg/kg in citrate buffer; Sigma, St. Louis, MO, USA) was given through intraperitoneal injection to induce type 2 diabetes mice. Tail vein blood was collected one week post injection. Mice with blood glucose levels > 16.7 mmol/L for consecutive 3 days were considered as diabetic onset and selected for further study [25].

To establish a wound healing model, after one month of diabetes induction, the dorsal fur was shaved the day before the operation and mice were anesthetized by pentobarbital sodium injection, then a round 1 cm area of skin was removed from the mouse's back to form a full-thickness wound. hADSC or hADSC^Hpgds^ were given at 5 × 10^5^ cells/ml mixed with VitroGel (TheWell Bioscience Company, North Brunswick, NJ, USA) at a ratio of 1:4 (v:v). All mice were randomly divided into three groups as follows (*n* = 35 each group): (1) PBS control group; (2) hADSC^PCDH^-VitroGel treated group; (3) hADSC^Hpgds^-VitroGel treated group. In addition, CM-Dil was used to label hADSC^Hpgds^ according to the manufacturer’s protocol (Invitrogen, USA), and the labeled hADSC^Hpgds^-VitroGel was detected by whole-body fluorescence imaging technique at 1 day, 3 days, 5 days, 7 days, 10 days, 14 days and 16 days (*n* = 5 each time point) to assess the safety of hADSC^Hpgds^.

### Morphometric analysis

At different time points (1, 3, 5, 7, 10, 14 and 17 days) after wounding, the wound photo was taken by a digital camera from a standard height. Wound area was measured by ImageJ software version 1.70 (National Institutes of Health, Bethesda, MD, USA). DW areas were quantified as follow: open wound rate (OWR) = open wound/ initial area of wound size [26]. The time was considered the healing day when the full-thickness wound was closed. Cross-sectional images of wound healing processes were captured under a light microscope (Leica DM3000, Wetzlar, Germany).

### Tissue preparation and the measurement of healing skin

At different time points we cut the tissues three millimeters around the wound edge for hematoxylin and eosin (HE), immunohistochemistry and immunofluorescence staining. The tissues within one millimeter to the wound edge were also obtained for qPCR detection. The thickness of healing skin including the epidermis and dermis were measured by ImageJ in HE stained sections. For statistical purpose, we randomly measured five locations on each section, and four non-consecutive sections were randomly selected in each wound. All measurements were performed independently by two independent observers who were blinded to group assignment.

### Immunofluorescence staining and immunohistochemistry

For immunofluorescence staining, the samples were incubated with antibodies (Abcam, Cambridge, UK) specific to proliferating cell nuclear antigen positive (PCNA, 1:200, #ab18197), F4/80 (1:200, #ab16911), and CD206 (1:200, #ab64693) overnight, followed by incubation with Alexa Fluor 488-goat anti-rabbit IgG (H + L) (1:200, #ab15007) and Alexa Fluor 594-goat anti-rat IgG (H + L) (1:200, #ab150160) for 1 h. For immunohistochemistry (IHC), the sections were incubated with CD31 (1:200, #ab28364, Abcam), collagen I (Col-I; 1:200, #ab34710, Abcam), Ly-6 g (1:200, #ab2577, Abcam), and CD8 (1:200, Abcam, #ab203035) antibodies overnight and then incubated with diaminobenzidine for 60 s and stained with hematoxylin for 60 s. Four non-consecutive sections were randomly selected in each wound. ImageJ was used to calculate the intensity of fluorescent signal and stained cells.

### Hpgds siRNA transfection in vivo

To evaluate the influence of *Hpgds* in normal wound, we prepared *Hpgds* siRNA in 1X siRNA buffer at the concentration of 1.5 g/ml. We intracutaneously injected 200 μl control siRNA or *Hpgds* siRNA (TsingKe, Beijing, China) around the wound bed every 2 days for 3 times. Wound edge tissue was harvested at 3 days after injection for histological detection. *Hpgds* siRNA sequences were as follows:

$$\begin{aligned} & {\text{F}}:{5}^{\prime }{\text{-}}\left( {{\text{mG}}} \right)\left( {{\text{mC}}} \right)\left( {{\text{mU}}} \right){\text{ACAUAUUCGCUUA}}\left( {{\text{mU}}} \right)\left( {{\text{mU}}} \right)\left( {{\text{mU}}} \right){\text{TT-3}}^{\prime } \\ & {\text{R}}:{5}^{\prime }{\text{-}}\left( {{\text{mA}}} \right)\left( {{\text{mA}}} \right)\left( {{\text{mA}}} \right){\text{UAAGCGAAUAUGU}}\left( {{\text{mA}}} \right)\left( {{\text{mG}}} \right)\left( {{\text{mC}}} \right){\text{TT-3}}^{\prime } . \\ \end{aligned}$$

### Culture of RAW 264.7 macrophages and LPS induction

The murine-derived macrophage cell line RAW 264.7 was provided by national biomedical laboratory cell repository (BMCR, Beijing, China). RAW 264.7 cells were cultured in Dulbecco’s Modified Eagle Medium (DMEM, HyCLone, Logan, Utah, USA) supplemented with 10% foetal bovine serum (FBS, Thermo Scientific, Waltham, Massachusetts, USA). The cells were passaged at approximately 80% confluence and were seeded in a 96-well tissue culture plate at a density of 2000 cells per well. After 24 h of incubation, the culture medium was removed and replaced by DMEM containing 10% FBS and LPS (200 ng/ml). After 12 h LPS induction, the medium was changed to the conditioned medium of hADSC^Hpgds^ and DMEM medium supplemented with PGD_2_ (50 μM) for another 24 h.

### The process and the primers of PCR

Total RNA was isolated from wound tissue samples using TRIzol reagent (Thermo fisher, USA) and reverse-transcribed by M-MLV reverse transcriptase (Promega, Madison, WI, USA). Quantitative real-time polymerase chain reaction (qRT-PCR) was performed on a LightCycler 480 system (Roche, Basel, Switzerland). Glyceraldehyde 3-phosphate dehydrogenase was used as the internal control. Primer sequences of the detected genes were listed in Additional file 1: Table S1.

### Statistical analysis

Data are presented as mean ± SD. Student’s *t*-tests were conducted for comparisons between two groups, and one-way analysis of variance was performed to assess statistical significance among multiple groups. Data were analyzed using SPSS 18 (SPSS Inc., Chicago, IL, USA). *P* < 0.05 was considered statistically significant.

## Results

### HPGDS is decreased in DW and its deficiency delays normal wound healing

To explore the comprehensive molecular characterization that leads to DW development, we analyzed differentially expressed genes between human DFS and NFS. The microarrays information was from Gene Expression Omnibus (GEO) database. The bioinformatics analysis showed that expression levels of 43 genes were different in DFS compared with NFS: 21 were upregulated, and 22 downregulated (Additional file 1: Table S2).

**Fig. 1:**
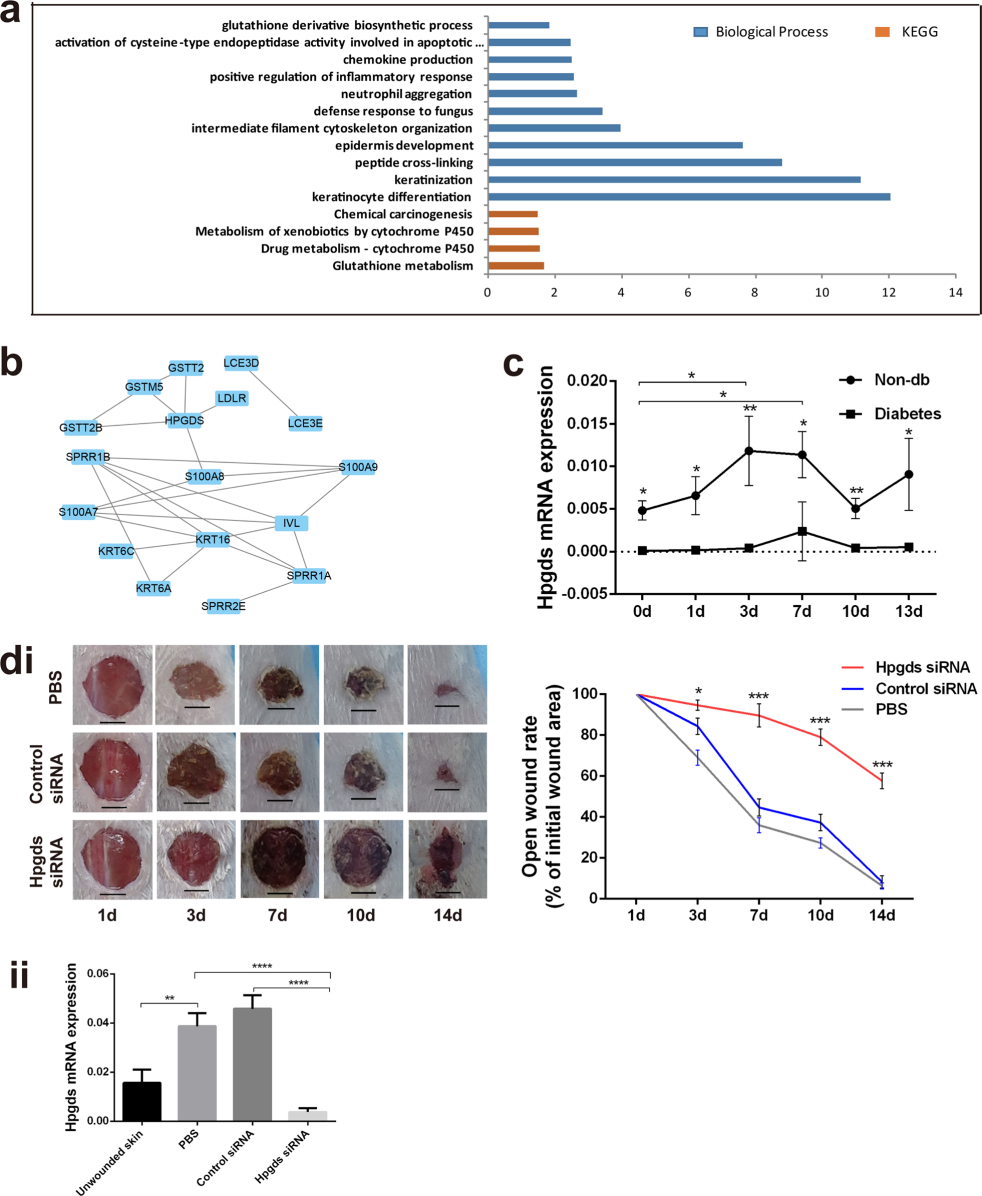
Expression of HPGDS decreases in diabetic wound bed and deficiency of Hpgds delays wound healing. **a** Gene Ontology analysis of transcriptome data from Gene Expression Omnibus database. **b** Interaction network of differential expression. **c** Dynamic *Hpgds* mRNA expression in wound edge tissue from diabetic mice at 0, 1, 3, 7, 10 and 13 days. The data are presented as the mean ± SD. *P < 0.05; **P < 0.01. *n* = 3. Abbreviation: non-db, nondiabetic mice; d, day. **d** Representative images of the gross appearance of non-db excisional wounds with PBS, control siRNA or *Hpgds* siRNA (i). Scale bar = 0.5 cm, *n* = 5. *Hpgds* mRNA expression in wound edge tissue was detected at 3 days by qRT-PCR (ii). *n* = 5. The data are presented as the mean ± SD. **P < 0.01; ****P < 0.0001. *CTL* Control, means normal mice without wound, *PBS* Phosphate buffer saline

Next, Gene Ontology (GO) and pathway analyses were performed (Fig. 1a), and we identified the GO terms concerning glutathione derivative biosynthetic process, positive regulation of inflammatory response, epidermis development, keratinization, and keratinocyte differentiation. On this basis, an interaction network was developed to show the relationships between differentially expressed proteins (Fig. 1b). The candidate target HPGDS located in a relatively central position, showing wide connections with other proteins. HPGDS was one of the downregulated genes and played an important role in immune regulation. To confirm the correlation of *Hpgds* with DW, we carried out *Hpgds* expression analysis in wound edge tissue at different time points (0, 1, 3, 7, 10, 13 days). And we observed that its expression was significantly reduced in type 2 diabetic mice compared to that in non-diabetic mice. During the healing process of normal mice wounds, *Hpgds* in the wounds were significantly increased on days 3 and 7 (*P* < 0.05, Fig. 1c). Moreover, in non-diabetic wound *Hpgds* expression increased in the first three days and maintained at a high level, then decreased after one week.

In addition, *Hpgds* siRNA was topically used in normal wound to elucidate whether *Hpgds* is involved in delayed wound healing. In the *Hpgds* siRNA treatment group, *Hpgds* expression in skin wound was significantly decreased compared with that in the control and control siRNA groups at 3 days (*P* < 0.05, Fig. 1dii), and skin wound healing was also significantly delayed compared with that in the control groups (*P* < 0.05, Fig. 1di). The results indicated that *Hpgds* deficiency significantly impeded wound healing.

To determine whether *Hpgds* played an anti-inflammatory role, the changes of *Hpgds* expression and its production were tested in RAW 264.7 macrophages after treatment with lipopolysaccharide. The results showed that the expressions of *Hpgds* (Additional file 1: Fig. S1a) and *cyclooxygenase-2* (Additional file 1: Fig. S1b), and the amount of PGD_2_ in LPS-induced macrophage supernatant were all significantly increased (Additional file 1: Fig. S1c). This suggested that *Hpgds* expression is elevated to modulate the inflammatory response.

### The inflammatory state is postponed in DW

**Fig. 2:**
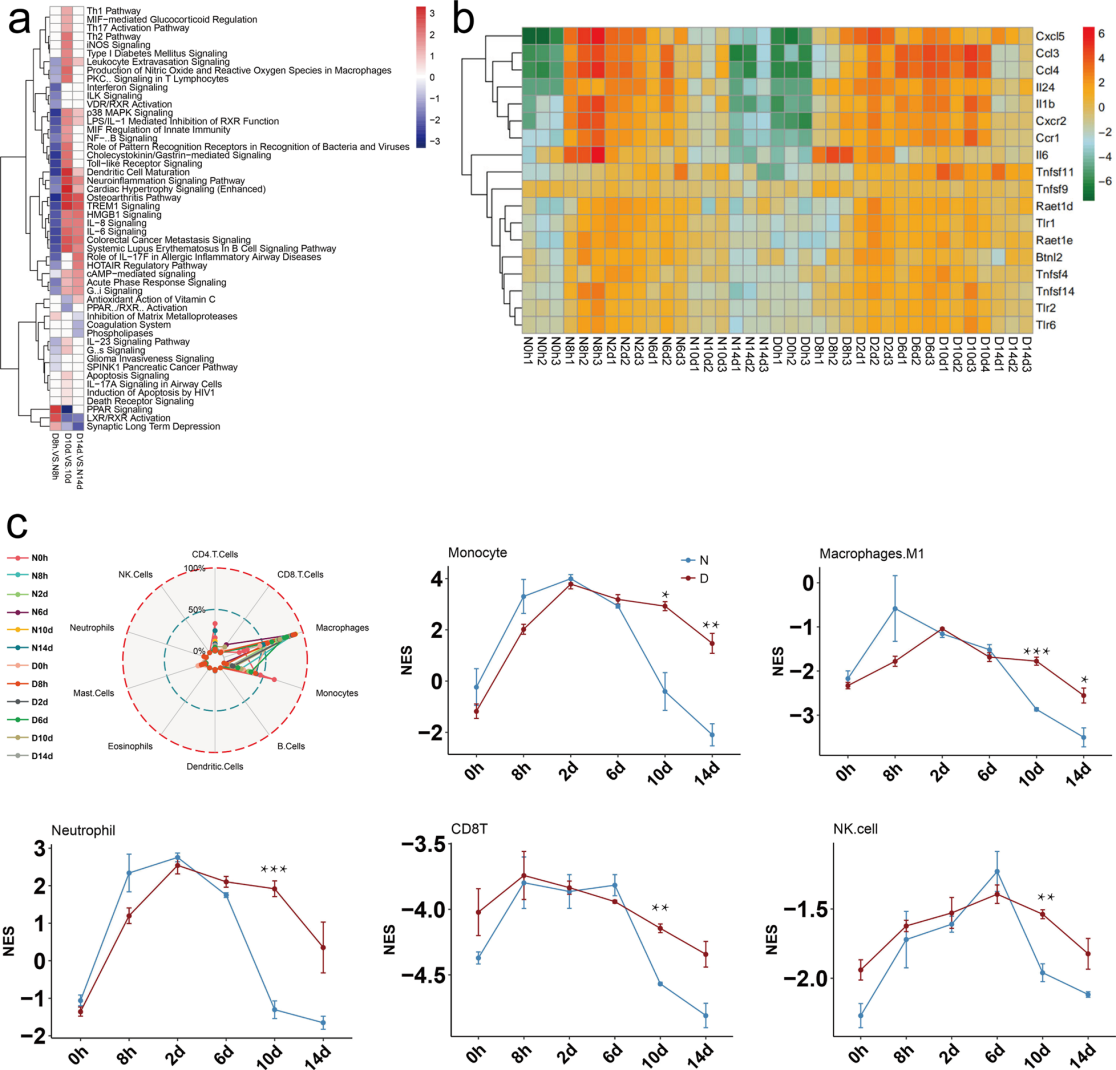
Characteristics of immune microenvironment in diabetic wound. **a** Heat map of immune pathway in db wound compared with that in non-db wound at 8 h, 10 days and 14 days. **b** Heat map of important immune factors in non-db/db wound at 0 h, 8 h, 2 days, 6 days, 10 days, and 14 days. **c** Proportion of main immune cells in non-db/db wound at 0 h, 8 h, 2 days, 6 days, 10 days, and 14 days. The data are presented as the mean ± SD. *P < 0.05; **P < 0.01; ***P < 0.001. *N* Non-diabetic mice group, *D* Diabetic mice group

To further understand the immune microenvironment in DWs, the comprehensive immune characteristics at different time points were determined. A heat map of the immune pathway showed that the activities of most immune-related pathways were lower in DWs at 8 h and higher at 10 days and 14 days compared with control wounds, such as TREM1 signaling, IL-6 signaling and acute phase response signaling (Fig. 2a). The heat map of immune factors showed that expressions of pro-inflammatory factors were also lower at 8 h and higher at 10 days and 14 days compared to the normal group, such as Cxcl5, Ccl3 and IL-1β (Fig. 2b).

Besides, ten major types of immune cells were present in the wound, and the top four cell types in number were macrophages, monocytes, CD4T and CD8T. Time course analysis showed that the proportion of pro-inflammatory cells and immune cells, such as monocyte, M1 macrophage, neutrophil, CD8T and NK cell, were significantly higher at 10 days in DW than that in normal wound (Fig. 2c). This suggested that the pro-inflammatory and immune responses in the wound of diabetic mice were persisted longer, which might contribute to the delayed DW healing.

### hADSC^Hpgds^ seeded into VitroGel accelerate DW healing

**Fig. 3:**
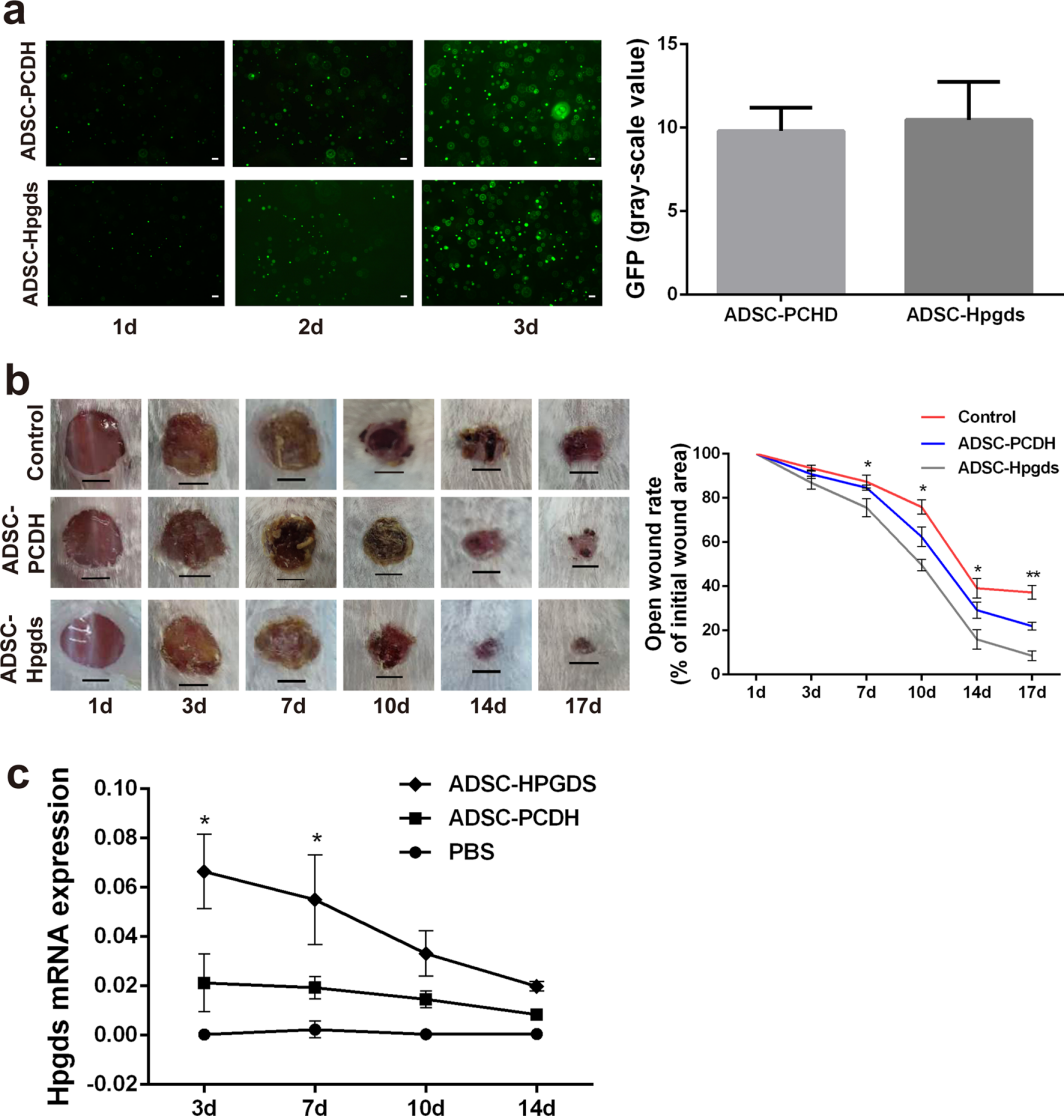
hADSC^Hpgds^ seeded in VitroGel 3D-RGD accelerates diabetic wound healing. **a** In vitro analysis of hADSC^Hpgds^ proliferation in VitroGel 3D-RGD at 1, 2, 3 days. *P* > 0.05, scale bar = 200 μm, *n* = 3. **b** Photographs of wound at 1, 3, 7, 10, 14 and 17 days incontrol, hADSC^PCDH^ and hADSC^Hpgds^ groups. Scale bar = 0.5 cm, *n* = 5. Open wound rate of initial wound area was analyzed. **c** Analysis of *Hpgds* mRNA expression in wound edge tissues at 3, 7, 10, 14 days. The data are presented as the mean ± SD. *P < 0.05, **P < 0.01. d, day

In hADSCs transduced with the PCDH-Hpgds vector, *Hpgds* expression was significantly increased by ~ 2000 times (*P* < 0.05) (Additional file 1: Fig. S2ai). And PGD_2_, produced by HPGDS, was 700 times higher in hADSC^Hpgds^ supernatant than in hADSC (*P* < 0.001) (Additional file 1: Fig. S2aii). Their biological characteristics showed no difference between hADSC^Hpgds^ and hADSC (Additional file 1: Fig. S2b-f). Then, the cells were mixed with VitroGel 3D-RGD that was highly biocompatible with seed cells (Fig. 3a). Time course area analysis showed that wound closure was significantly accelerated in the hADSC^Hpgds^-VitroGel treated group from 7 to 17 days compared with hADSC^PCDH^-VitroGel treated group and control group (Fig. 3b).

*Hpgds* expression was analyzed in the wound tissue after treatment with the transduced MSCs. The results showed that the expression of *Hpgds* in the hADSC^Hpgds^ group was significantly higher than that in the empty vector and PBS groups at 3 days and 7 days after treatment in DW (Fig. 3c).

To assess the safety of hADSC^Hpgds^ treatment, in vivo tracking and immunofluorescence sections of the wound tissue showed that CM-Dil fluorescence signal was confined to the wound without distant metastasis (Additional file 1: Fig. S3ai, aii). Hematoxylin–eosin staining showed no difference in multiple tissues between hADSC^Hpgds^-VitroGel treated mice and normal mice (Additional file 1: Fig. S3b).

### hADSC^Hpgds^ promotes proliferation and vascularization in DW

**Fig. 4:**
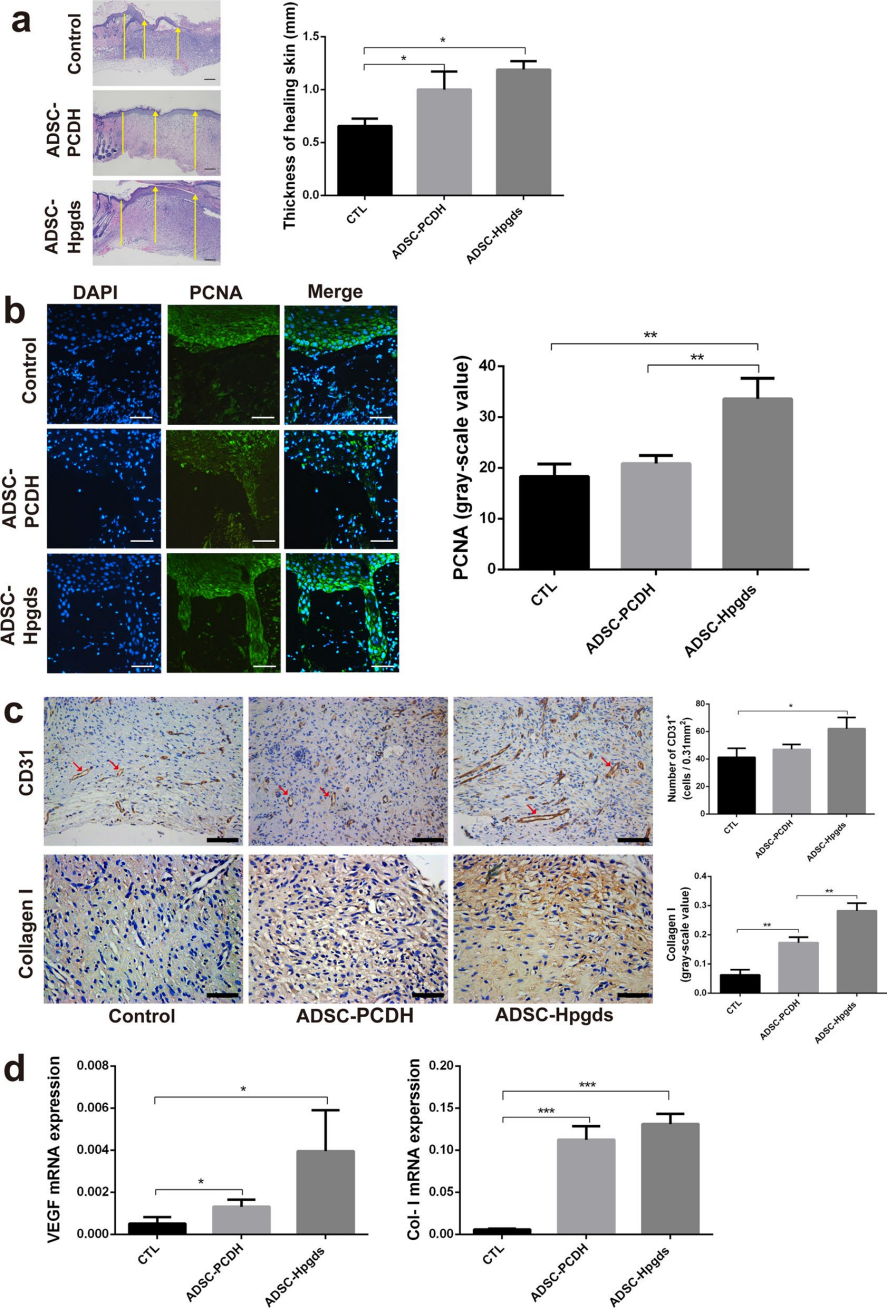
hADSC^Hpgds^ promotes proliferation and vascularization. **a** Thickness of the healing skin. Hematoxylin and eosin staining for the sections of wound edge tissue at 14 days, *n* = 5. Yellow line indicates the healing edge; yellow arrows indicate the thickness of the healing skin including the epidermis and dermis. Scale bar = 200 μm. **b** Immunofluorescence staining of PCNA in the healing skin within one millimeter to the healing edge at 10 days. Blue, DAPI; green, PCNA; Average area of positive fluorescence per group was quantified. Scale bar = 100 μm. **c** Immunohistochemical staining for CD31 and Collagen-I in the healing skin within one millimeter to the healing edge at 14 days; Average number of positive cells and gray-scale value per group were quantified. Red arrows indicate vascular endothelial cells. Scale bar = 100 μm. **d** The mRNA expressions of VEGF and Collagen-I in the healing skin were quantified at 14 days. The data are presented as the mean ± SD. *P < 0.05; **P < 0.01; ***P < 0.001. *n* = 5. *CTL* Control, means treatment with PBS

Hematoxylin–eosin staining of wound tissues were performed 14 days after surgery and showed that the healing skin was thicker in hADSC^Hpgds^ and hADSC^PCDH^ groups compared with control group (*P* < 0.05, Fig. 4a). Cell proliferation in the healing skin were analyzed at 10 days after surgery, and significantly more PCNA^+^ cells presented in the hADSC^Hpgds^ group than in control groups (*P* < 0.05, Fig. 4b).

Moreover, compared to the control, the hADSC^Hpgds^ group had significantly higher numbers of CD31^+^ endothelial cells and more scattered small blood vessels at 14 days (Fig. 4c), indicating increased angiogenesis and vascularity. The gray-scale value of Col-I in hADSC^Hpgds^ group was also significantly higher than in the control groups (*P* < 0.05). The IHC results were confirmed by significantly increased mRNA expressions of vascular endothelial growth factor (*VEGF*) and *Col-I* in hADSC^Hpgds^ group (*P* < 0.05, Fig. 4d).

### hADSC^Hpgds^ modulates the inflammatory state in DW

**Fig. 5:**
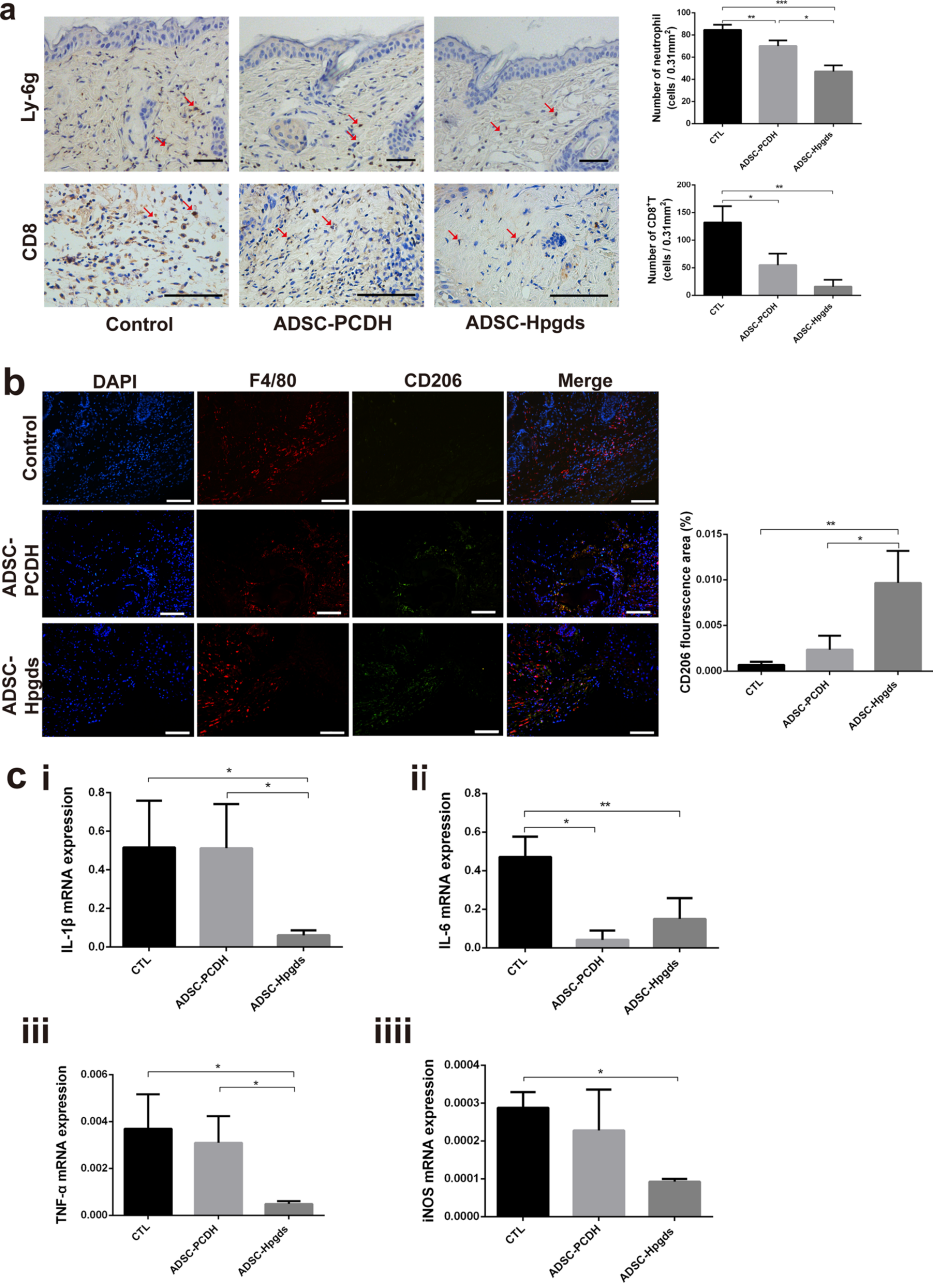
hADSC^Hpgds^ regulates inflammatory environment. **a** Immunohistochemical stainings for Ly-6 g and CD8 in the tissue surrounding the wound at 3 days. Average number of positive cells per group was quantified. Red arrows indicate positive staining cells. **b** Immunofluorescence of F4/80, CD206 (Blue, DAPI; red, F4/80; green, CD206) in the healing skin within one millimeter to the healing edge at 7 days. Average area of positive fluorescence per group was quantified. **c** IL-1β (i), IL-6 (ii), TNF-α (iii) and iNOS (iiii) mRNA expressions were quantified at 7 days. The data are presented as the mean ± SD. *P < 0.05; **P < 0.01; ***P < 0.001. *n* = 5. *CTL* Control, means treatment with PBS; a, b scale bar = 100 μm

IHC for neutrophils and CD8T cells in the tissue surrounding the wound at 3 days showed weaker signals with significantly decreased numbers of neutrophils and CD8T cells in hADSC^Hpgds^ group than in hADSC^PCDH^ and control groups (Fig. 5a). In contrast, significantly more F4/80^+^CD206^+^ M2 macrophages were observed in the healing skin in hADSC^Hpgds^ group at 7 days (Fig. 5b), and mRNA expression levels of the inflammatory factors tumor necrosis factor (TNF)-α, IL-6, interleukin (IL)-1β, and induced nitric oxide synthase (iNOS) were significantly decreased in hADSC^Hpgds^ group (*P* < 0.05, Fig. 5c).

### hADSC^Hpgds^ conditioned medium and PGD_2_ promote M2 polarization in vitro

**Fig. 6:**
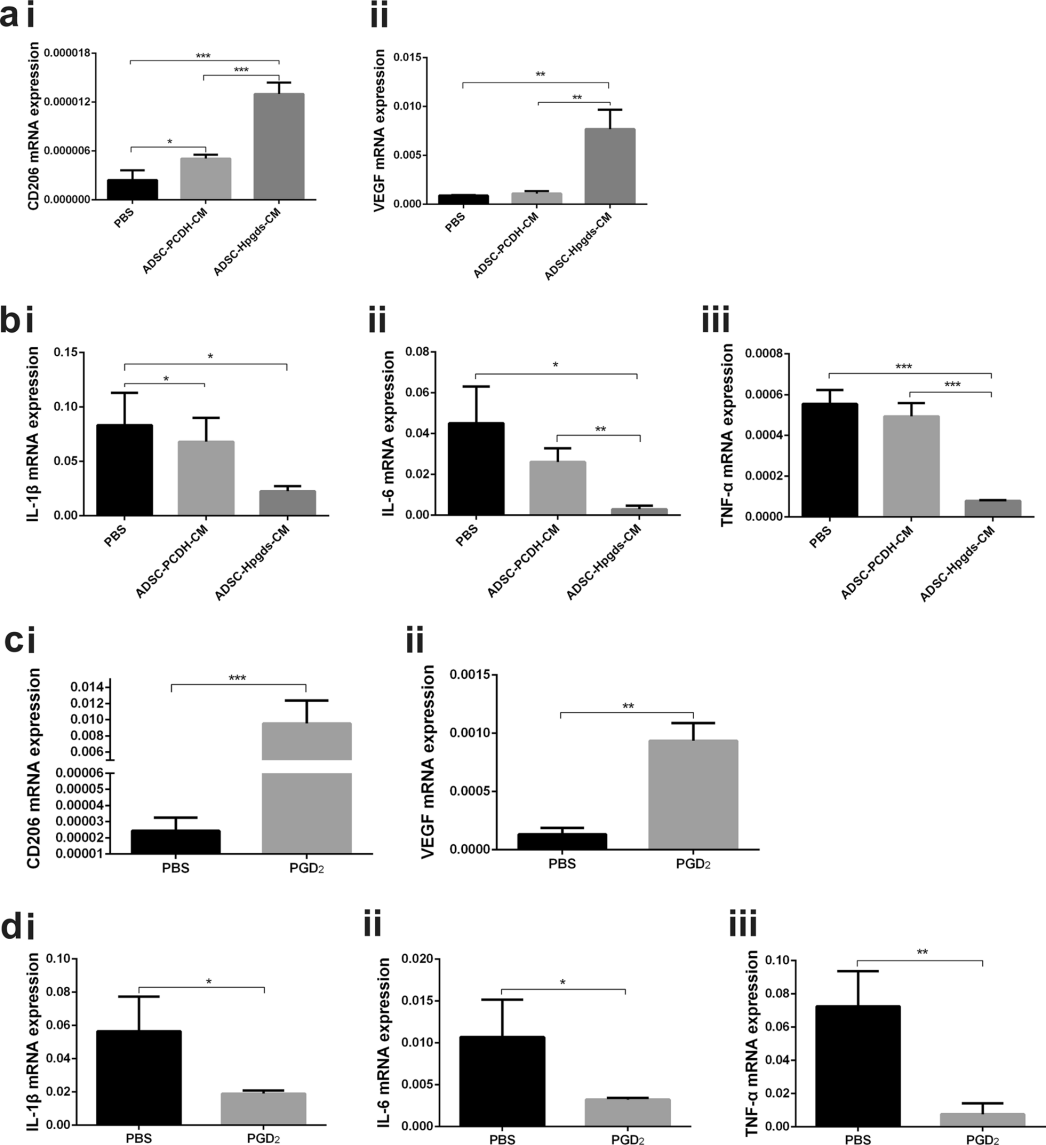
hADSC^Hpgds^ conditional medium or PGD_2_ promotes M2 polarization. **ai** Expression of CD206 in LPS-induced macrophage after treated with hADSC^Hpgds^ CM for 24 h was detected by qRT-PCR. VEGF (**aii**) and IL-1β (**bi**), IL-6 (**bii**) and TNF-α (**biii**) mRNA expressions in LPS-induced macrophage were quantified by qRT-PCR. mRNA expressions of CD206 (**ci**), VEGF (**cii**), IL-1β (**di**), IL-6 (**dii**) and TNF-α (**diii**) in LPS-induced macrophage after treated with PGD_2_ (50 μM) for two days was detected by qRT-PCR. The data are presented as the mean ± SD. *P < 0.05; **P < 0.01; ***P < 0.001. *n* = 4. *PBS* Phosphate buffer saline

To assess paracrine effects of hADSC^Hpgds^ on macrophage, the conditioned medium (CM) was used to culture LPS-induced RAW 264.7 macrophage. qRT-PCR analysis showed that CD206 and VEGF expression were significantly increased after culturing in hADSC^Hpgds^ CM than in hADSC CM (*P* < 0.001, Fig. 6ai–ii), moreover, expression levels of inflammatory factors TNF-α, IL-6 and IL-1β were significantly decreased in hADSC^Hpgds^ CM group than in hADSC CM and PBS groups (*P* < 0.05, Fig. 6bi–iii), indicating superior capability of hADSC^Hpgds^ CM for promoting M2 macrophage polarization.

Furthermore, we explored whether there were similar effects of PGD_2_ on LPS-induced macrophages in vitro. CD206 expression was dramatically increased after adding PGD_2_ (50 μM) to culture medium for 24 h compared to control (*P* < 0.001, Fig. 6ci) with a larger quantity series than hADSC^Hpgds^ CM treatment. VEGF expression in LPS-induced macrophages was also significantly higher in PGD_2_ group than in control group (*P* < 0.01, Fig. 6cii), whereas IL-1β, IL-6, and TNF-α levels were significantly lower than in control group (*P* < 0.05, Fig. 6di–iii).

## Discussion

DWs carry risks of infection, amputation, and high morbidity [27]. They are the leading cause of non-traumatic amputation of lower limbs [28], and patients who require an amputation have a 5-year mortality risk of 74% [29]. Our study demonstrates that *Hpgds* deficiency is a critical factor that impedes cutaneous wound healing in diabetes. Additionally, we found that in mice DW immune pathways, immune cell components, and immune molecules expression were suppressed in the early stage and aggravated in later stages. Engineered hADSCs overexpressing *Hpgds* accelerated the skin wound healing process in type 2 diabetic mice through improving tissue proliferation, vascularization, and the anti-inflammatory state by increasing M2 polarization and reducing neutrophil and CD8T cell recruitment. Finally, in vitro experiments showed conditional medium of hADSC^Hpgds^ and adequate PGD_2_ secreted by hADSC^Hpgds^ had the ability to shift macrophages from pro-inflammatory to healing-associated phenotypes, which might be alternative strategies for DWs healing.

Delayed DW is characterized by persistent inflammation with excessive polarization of M1 macrophages, the recruitment of large numbers of neutrophils and increased inflammatory cytokine secretion [30]. Indeed, our data confirmed that in the late stage of DWs pro-inflammatory immune responses were severer than in normal wound, which was unfavorable for DWs healing turning from inflammation to proliferation stage. Moreover, we found that in the early inflammatory stage DW showed weaker activities of immune-related signaling pathways, decreased immune cells number and immune molecules expression, such as inhibited signaling pathways of NF-κB and IL-6, decreased numbers of monocytes, macrophages, and neutrophils in the wound, which were not advantageous to eliminate pathogenic microorganisms. Therefore, ameliorating the disturbed inflammation state is crucial for improving DW healing.

It is known that macrophage phenotype transition from pro-inflammatory (M1) to anti-inflammatory (M2) plays important roles during wound healing. M1 macrophages were over accumulated in diabetic ulcers, and secreted a lot of pro-inflammation factors, such as IL-6, TNF-α, iNOS [31]. M2 macrophages produced anti-inflammatory mediators and growth factors, such as arginase-1, IL-10, TGF-β and VEGF [32]. Many studies demonstrated abnormal M1-M2 transformation in DW; however, the mechanism has not been well defined. The upregulation of peroxisome proliferator-activated receptor gamma (PPAR-γ), a transcription factor actively participating in lipid metabolism, was reported to involve in macrophage polarization switch in normal wound, whereas it was impaired in DW [33]. In our study, we confirmed that *Hpgds* was downregulated in DW, and its deficiency delayed normal mice wound healing. It had been reported that HPGDS catalyzes PGH_2_ into anti-inflammatory factors PGD_2_/15d-PGJ_2_ [34, 35]. PGD_2_–DP1 axis-induced M2 polarization helps resolve inflammation through the separated PKA regulatory IIα subunit (PRKAR2A)-mediated suppression of JAK2/STAT1 signaling [18]. 15d-PGJ2 also exerts antiproliferative and cytotoxic effects on lymphocytes and macrophages by activating PPAR-γ [36]. These studies indicated that prostaglandin production could be novel target for metabolically associated inflammatory complications. Our results demonstrated that *Hpgds* plays a key role in the wound healing process through inducing M1-M2 transition. It could be an efficient strategy for coordinating macrophage polarization and improving healing of DW.

ADSC therapy has been regarded as a promising strategy for promoting wound healing mainly due to anti-inflammatory effects and proangiogenic production [2, 37–39]. Moreover, exosomes secreted by ADSCs and anti-inflammatory cytokines have also been reported to significantly accelerate the wound healing process [40]. Considering the complexity of diabetic wounds, in our study we overexpressed Hpgds in hADSCs encapsulated by VitroGel 3D-RGD, which strategy strengthened the immunomodulatory properties of ADSCs. Except for decreased M1 and increased M2 macrophages in hADSC^Hpgds^ group, the reduction of neutrophils and CD8T cells was also observed. Moreover, in hADSC^Hpgds^ group the higher expression of angiogenesis-stimulating factors VEGF and CD31 could be resulted from Hpgds-induced M1-M2 transition [41], as well as the increased collagen I which biosynthesis might be regulated by the major stimulator TGF-β secreted by M2 macrophage [42]. Furthermore, more hair follicles in the wound edge tissue were found in hADSC and hADSC^Hpgds^ groups, which was consistent with the report that ADSCs conditioned medium stimulated the growth of human dermal papilla cells and the elongation of hair shafts in isolated hair follicles [43], and might potentially contribute to the re-epithelialization and vascularization in wound healing [44]. Our results showed a better repair for diabetic wound in hADSC^Hpgds^ group than in hADSC alone group, accompanied by more proper inflammation state without side effects.

Moreover, we found that the ADSC^Hpgds^ CM could effectively regulate the LPS-induced inflammation by increasing M2 polarization and decreasing the expression of pro-inflammatory cytokines IL-1β, IL-6 and TNF-α in LPS-induced macrophages, indicating that *Hpgd*s enhanced the immune regulation ability of ADSCs in vitro. Additionally, the function of small molecular PGD_2_ on LPS-induced macrophages was analyzed and presented similar effects as ADSC^Hpgds^ CM. Indeed, these data only support the influences of HPGDS-PGD_2_ on macrophages. The in vitro experiments of *Hpgds* effects on other inflammatory cells related to diabetic wounds could provide more evidence for their roles. Besides, the limitation of our study was that the underlying molecular mechanisms why *Hpgds* expression decreases in diabetic wounds and how it leads to prolonged and unhealed DW need to be further addressed.

## Conclusion

This study provides evidence that delayed DW healing is caused by persistent inflammation and a failure to transform into the proliferation phase, which is associated with *Hpgds* downregulation. Overexpressing *Hpgds* in ADSCs could be an effective way to accelerate DW healing by alleviating inflammatory cells recruitment and increasing M2 polarization in the proliferation phase. In the future, in vivo experiments of ADSC^Hpgds^ conditioned medium or small molecular PGD_2_ on diabetic wound could be performed to study whether they could be alternative clinical strategies.

## Supplementary Information

**Additional file 1. Table S1.** Oligonucleotide primers used in the real-time. **Table S2.** Differentially expressed genes between non-diabetic and diabetic full thickness skin biopsies. **Fig. S1.** Hpgds and COX-2 were significantly increased in LPS-induced RAW 264.7 macrophage. **a**–**c** Hpgds (**a**) and COX-2 (**b**) mRNA expression in RAW 264.7 macrophage treated with LPS (200 ng/ml) for 12 h were detected by qRT-PCR and PGD_2_ (**c**) concentration in culture medium was detected by elisa. The data are presented as the mean ± SD. *P < 0.05; **P < 0.01. *n* = 5. **Fig. S2.** Hpgds overexpression and its influence on hADSC biological characeristics. **a** Hpgds mRNA expression efficiency in hADSCHpgds (i); Concentration of PGD2 in supernatant of hADSCHpgds (ii). **b** Flow cytometry analysis of stem cells markers, CD44, CD73, CD90 and CD105 on hADSCHpgds. *P* > 0.05. **c** Proliferation ability of hADSCHpgds at 1, 2, 3, 4, 5, 6 and 7 days. *P* > 0.05. **d** hADSCHpgds apoptosis analysis. *P* > 0.05. **e** hADSCHpgds migration analysis at 0, 6, 12, 24 h. *P* > 0.05. **f** hADSCHpgds adipogenic and osteogenic differentiation staining at 2 weeks and 3 weeks, respectively, and PPARγ and RUNX2 mRNA expression analysis at 7 days and 14 days. *P* > 0.05. The data are presented as the mean ± SD. *n* = 5. **Fig. S3.** In vivo tracking and security of hADSC^Hpgds^. **a** Imagings were taken after hADSC^Hpgds^-VitroGel transplanted at wound bed (i) and wound edge tissue slice images (ii) at 1, 3, 5, 7, 10, 14 and 16 days. The data are presented as the mean ± SD. *n* = 5. **b** Hematoxylin-eosin staining analysis of the skin and muscle around wound and heart, liver, spleen, lung and kidney 3 months postsurgery.

## Data Availability

The datasets used and/or analyzed during the present study are available from the corresponding author on reasonable request.

## Abbreviations

DW: Diabetic wound
HPGDS: Hematopoietic prostaglandin D synthase
ADSC: Adipose-derived mesenchymal stem cell
DFS: Diabetic human foot skin
NFS: Non-diabetic human foot skin
siRNA: Short-interfering RNA
PGH_2_: Prostaglandin H2
PGD_2_: Prostaglandin D2
GEO: Gene expression omnibus
GO: Gene ontology
