# Supplementary material for: Overexpressing HPGDS in adipose-derived mesenchymal stem cells reduces inflammatory state and improves wound healing in type 2 diabetic mice
Source: Stem Cell Res Ther. 2022 Aug 3;13:395. doi: 10.1186/s13287-022-03082-w (PMC9351105; doi:10.1186/s13287-022-03082-w)
Supplement: Supplementary file 1 — Additional file 1. Table S1. Oligonucleotide primers used in the real-time. Table S2. Differentially expressed genes between non-diabetic and diabetic full thickness skin biopsies. Fig. S1. Hpgds and COX-2 were significantly increased in LPS-induced RAW 264.7 macrophage. a–c Hpgds (a) and COX-2 (b) mRNA expression in RAW 264.7 macrophage treated with LPS (200 ng/ml) for 12 h were detected by qRT-PCR and PGD2 (c) concentration in culture medium was detected by elisa. The data are presented as the mean ± SD. *P < 0.05; **P < 0.01. n = 5. Fig. S2. Hpgds overexpression and its influence on hADSC biological characeristics. a Hpgds mRNA expression efficiency in hADSCHpgds (i); Concentration of PGD2 in supernatant of hADSCHpgds (ii). b Flow cytometry analysis of stem cells markers, CD44, CD73, CD90 and CD105 on hADSCHpgds. P > 0.05. c Proliferation ability of hADSCHpgds at 1, 2, 3, 4, 5, 6 and 7 days. P > 0.05. d hADSCHpgds apoptosis analysis. P > 0.05. e hADSCHpgds migration analysis at 0, 6, 12, 24 h. P > 0.05. f hADSCHpgds adipogenic and osteogenic differentiation staining at 2 weeks and 3 weeks, respectively, and PPARγ and RUNX2 mRNA expression analysis at 7 days and 14 days. P > 0.05. The data are presented as the mean ± SD. n = 5. Fig. S3. In vivo tracking and security of hADSCHpgds. a Imagings were taken after hADSCHpgds-VitroGel transplanted at wound bed (i) and wound edge tissue slice images (ii) at 1, 3, 5, 7, 10, 14 and 16 days. The data are presented as the mean ± SD. n = 5. b Hematoxylin-eosin staining analysis of the skin and muscle around wound and heart, liver, spleen, lung and kidney 3 months postsurgery. [file 13287_2022_3082_MOESM1_ESM.docx]

| **Supplementary table 1.** Oligonucleotide primers used in the real-time RT-PCR. | |
| --- | --- |
| Gene | Sequence (5’-3’) |
| Hpgds | F: 5'-GGAAGAGCCGAAATTATTCGCT-3' |
|  | R: 5'-ACCACTGCATCAGCTTGACAT-3' |
| COX2 | F: 5'-TGTGACTGTACCCGGACTGG-3' |
|  | R: 5'-GCACATTGTAAGTAGGTGGAC-3' |
| RUNX2 | F: 5'-ACTGGCGCTGCAACAAGAC-3' |
|  | R: 5'-CCCGCCATGACAGTAACCA-3' |
| VEGF | F: 5'-GTCCCATGAAGTGATCAAGTTC-3' |
|  | R: 5'-TCTGCATGGTGATGTTGCTCTCTG-3' |
| Col-I | F: 5'-CAGGCAAACCTGGTGAACA-3' |
|  | R: 5'-CTCGCCAGGGAAACCTCT-3' |
| IL-1β | F: 5'-TGGAGAGTGTGGATCCCAAG-3' |
|  | R: 5'-GGTGCTGATGTACCAGTTGG-3' |
| IL-6 | F: 5'-ATAGTCCTTCCTACCCCAATTTCC-3' |
|  | R: 5'-GATGAATTGGATGGTCTTGGTCC-3' |
| TNF-α | F: 5'-CTGAACTTCGGGGTGATCGG-3' |
|  | R:5'-GGCTTGTCACTCGAATTTTGAGA-3' |
| iNOS | F: 5'-GTTCTCAGCCCAACAATACAAGA-3' |
|  | R: 5'-GTGGACGGGTCGATGTCAC-3' |

**Supplementary table 1-** Primer sequences that used in the real-time PCR were listed in the table.

| **Supplementary table 2**. Differentially expressed genes between non-diabetic and diabetic full thickness skin biopsies  (FC, Fold Change) | | | | | |
| --- | --- | --- | --- | --- | --- |
| Gene Symbol | Log_2_FC | P.Value | Gene Symbol | Log_2_FC | P.Value |
| \| KRT6C \| \| --- \| \| S100A8 \| \| S100A9 \| \| KRT6A \| \| S100A7 \| \| KRT16 \| \| SPRR1A \| \| GSTT2 \| \| LCE3E \| \| SPRR1B \| \| GSTT2B \| \| MIR1302-5 \| \| LCE3D \| \| FETUB \| \| SPRR2E \| \| XDH \| \| LINC01139 \| \| IVL \| \| LDLR \| \| GDPD3 \| \| C10orf99 \| | \| 3.258847 \| \| --- \| \| 2.85795 \| \| 2.676497 \| \| 2.49616 \| \| 2.268463 \| \| 2.248343 \| \| 2.20926 \| \| 1.898147 \| \| 1.459703 \| \| 1.434957 \| \| 1.374563 \| \| 1.345367 \| \| 1.259 \| \| 1.20493 \| \| 1.17875 \| \| 1.121737 \| \| 1.086837 \| \| 1.06806 \| \| 1.03787 \| \| 1.022927 \| \| 1.01489 \| | \| 0.026869 \| \| --- \| \| 0.008765 \| \| 0.000495 \| \| 0.002073 \| \| 0.030858 \| \| 0.001237 \| \| 0.00994 \| \| 0.046399 \| \| 0.008566 \| \| 0.002037 \| \| 0.049836 \| \| 0.004801 \| \| 0.026693 \| \| 0.011175 \| \| 0.038795 \| \| 0.003551 \| \| 0.025089 \| \| 0.020629 \| \| 0.012994 \| \| 0.001179 \| \| 0.010985 \| | \| NRN1 \| \| --- \| \| LOC389765 \| \| HPGDS \| \| NKAPP1 \| \| MIR548I1 \| \| MIR548I2 \| \| LRRC2 \| \| FGL2 \| \| PMP22 \| \| MIR548I3 \| \| CP \| \| GPR174 \| \| MIR1245A \| \| TPSB2 \| \| PAMR1 \| \| THBS4 \| \| FNDC1 \| \| MIRLET7A2 \| \| GSTM5 \| \| ANGPTL1 \| \| LGR5 \| \| WIF1 \| | \| -1.00536 \| \| --- \| \| -1.02456 \| \| -1.03045 \| \| -1.0367 \| \| -1.04147 \| \| -1.05277 \| \| -1.05641 \| \| -1.05757 \| \| -1.06014 \| \| -1.09702 \| \| -1.14229 \| \| -1.16383 \| \| -1.26104 \| \| -1.29323 \| \| -1.31972 \| \| -1.34606 \| \| -1.39503 \| \| -1.51933 \| \| -1.59936 \| \| -1.637 \| \| -1.93939 \| \| -2.57026 \| | \| 0.002075 \| \| --- \| \| 0.001866 \| \| 0.031271 \| \| 0.024383 \| \| 0.010668 \| \| 0.010515 \| \| 0.022559 \| \| 0.013008 \| \| 0.048738 \| \| 0.022115 \| \| 0.036782 \| \| 0.021024 \| \| 0.029884 \| \| 0.00382 \| \| 0.031018 \| \| 0.03132 \| \| 0.004436 \| \| 0.000619 \| \| 0.000214 \| \| 0.013513 \| \| 0.001595 \| \| 0.005941 \| |

**Supplementary table 2** The bioinformatics analysis showed that expression levels of 43 genes were different in DFS compared with NFS: 21 were upregulated and 22 downregulated.


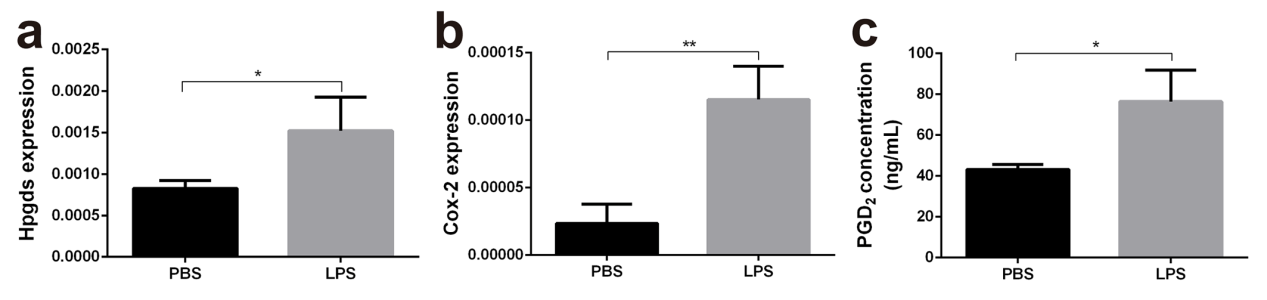


**Supplementary Fig. 1 Hpgds and COX-2 were significantly increased in LPS-induced RAW 264.7 macrophage**. a-c: Hpgds (a) and COX-2 (b) mRNA expression in RAW 264.7 macrophage treated with LPS (200 ng/ml) for 12 hours were detected by qRT-PCR and PGD_2_ (c) concentration in culture medium was detected by elisa. The data are presented as the mean ± SD. **P* < 0.05; ***P* < 0.01. n=5.

**
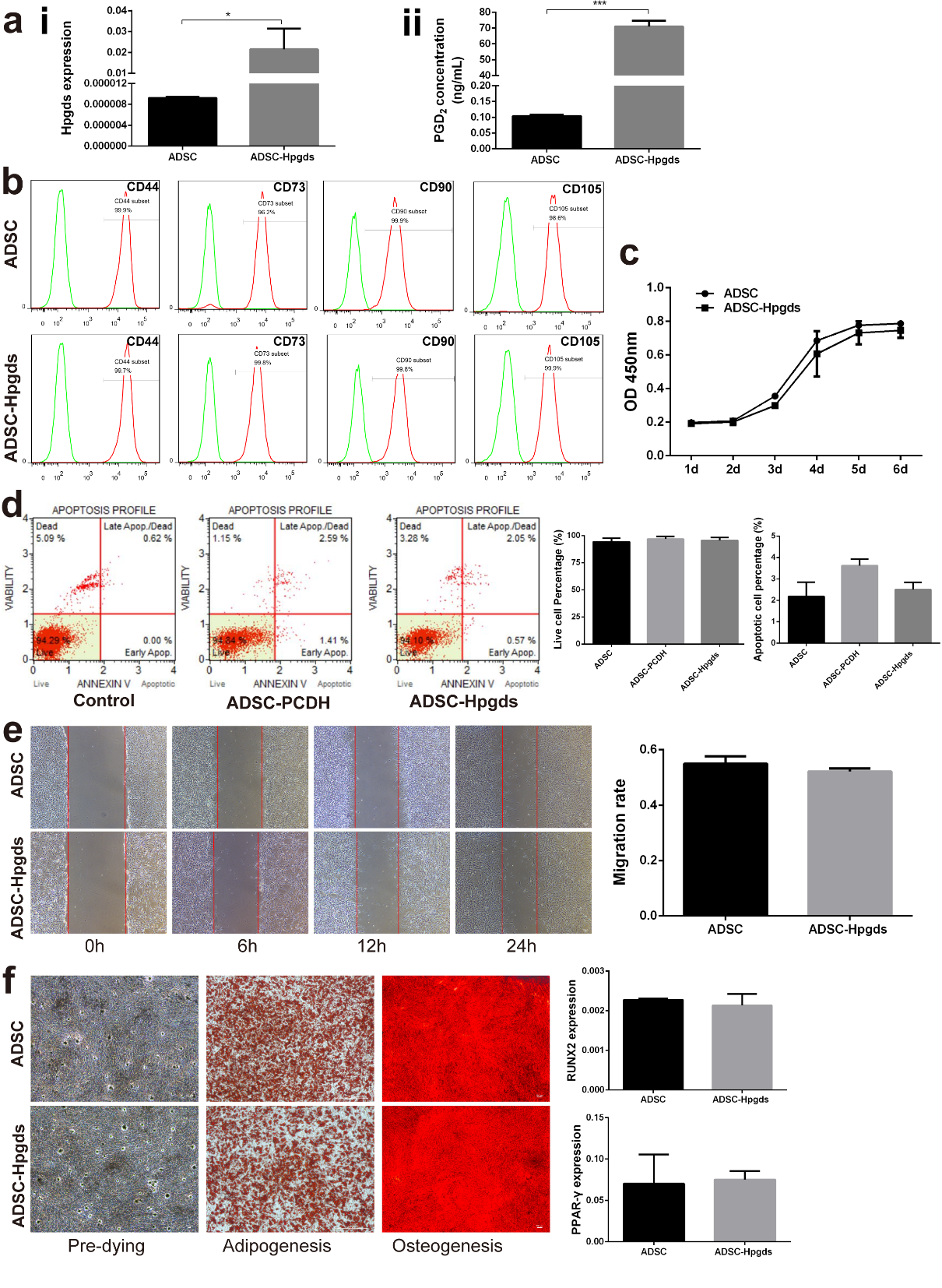
**

**Supplementary Fig. 2** **Hpgds overexpression and its influence on hADSC biological characeristics.** a: Hpgds mRNA expression efficiency in hADSC^Hpgds^ (i); Concentration of PGD_2_ in supernatant of hADSC^Hpgds^ (ii). b: Flow cytometry analysis of stem cells markers, CD44, CD73, CD90 and CD105 on hADSC^Hpgds^. *P*﹥0.05. c: Proliferation ability of hADSC^Hpgds^ at 1, 2, 3, 4, 5, 6 and 7 days. *P*﹥ 0.05. d: hADSC^Hpgds^ apoptosis analysis. *P*﹥0.05. e: hADSC^Hpgds^ migration analysis at 0, 6, 12, 24h. P﹥0.05. f: hADSC^Hpgds^ adipogenic and osteogenic differentiation staining at 2 weeks and 3 weeks, respectively, and PPARγ and RUNX2 mRNA expression analysis at 7 days and 14 days. *P*﹥0.05. The data are presented as the mean ± SD. n = 5.


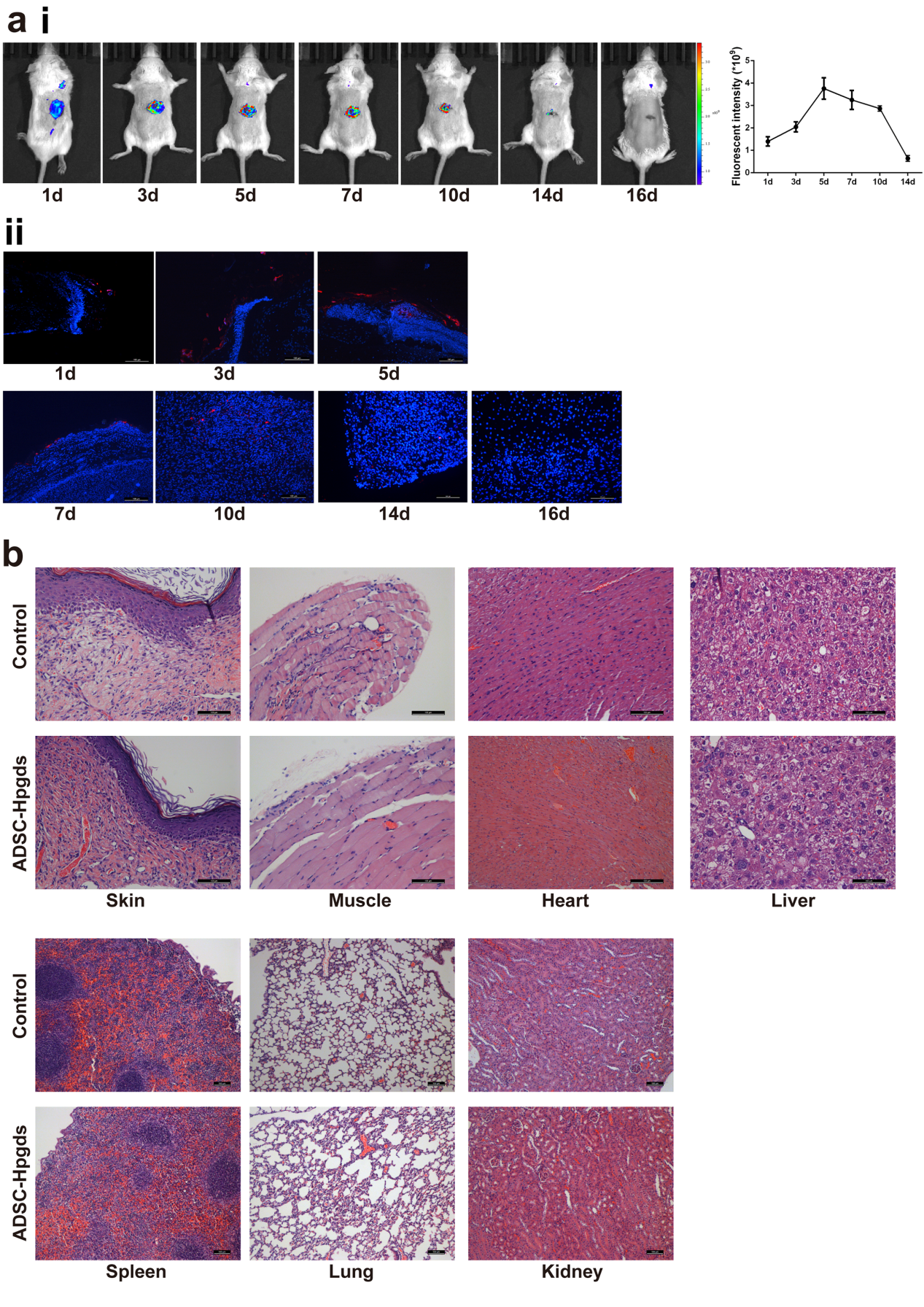


**Supplementary Fig. 3** **In vivo tracking and security of hADSC^Hpgds^**. a: Imagings were taken after hADSC^Hpgds^-VitroGel transplanted at wound bed (i) and wound edge tissue slice images (ii) at 1, 3, 5, 7, 10, 14 and 16 days. The data are presented as the mean ± SD. n = 5. b: Hematoxylin-eosin staining analysis of the skin and muscle around wound and heart, liver, spleen, lung and kidney 3 months postsurgery.
